# Prolonged Responses in Central Nervous System Relapsed Diffuse Large B-Cell Lymphoma After Chimeric Antigen Receptor T-Cell Therapy Using Targeted Treatments

**DOI:** 10.7759/cureus.66291

**Published:** 2024-08-06

**Authors:** Simon Planken, Sylvia Faict, Fabienne Trullemans, Eleni Linskens, Karl Vandepoele, Ann De Becker

**Affiliations:** 1 Department of Hematology, Universitair Ziekenhuis Brussel, Jette, BEL; 2 Department of Clinical Biology, Universitair Ziekenhuis Brussel, Jette, BEL; 3 Department of Clinical Biology, Universitair Ziekenhuis Gent, Gent, BEL

**Keywords:** btk inhibitor, treatment sequencing, cancer immunotherapy, chimeric antigen receptor t-cell therapy, diffuse large b-cell lymphoma

## Abstract

The introduction of chimeric antigen receptor T-cell (CAR-T cell) therapy has changed the treatment landscape of diffuse large B-cell lymphoma (DLBCL). However, the optimal treatment strategy after relapse after this therapy still needs to be elucidated. In this report, we describe the case of a 67-year-old male who relapsed after treatment with tisagenlecleucel as a third-line therapy. We present our approach to treatment after relapse, in which we tried to sustain the circulating chimeric antigen receptor T-cells. This is reflected by the kinetics of the chimeric antigen receptor T-cells during these treatments.

## Introduction

Diffuse large B-cell lymphoma (DLBCL) is the most common lymphoid neoplasm in the Western world [1]. The disease is characterized by an aggressive clinical presentation, with approximately two-thirds of patients presenting with advanced disease [2]. The mainstay of treatment has been rituximab combined with chemotherapy, with the preferred regimen being rituximab, cyclophosphamide, daunorubicin, vincristine, and prednisone (R-CHOP). Recently, an improved response rate and progression-free survival (PFS) have been demonstrated by replacing vincristine with polatuzumab vedotin [3]. However, 10% of patients do not respond sufficiently to first-line treatment, and around one-third of patients will relapse after attaining a complete response [2]. In patients with relapsed or refractory DLBCL (R/R DLBCL), treatment with chimeric antigen receptor T-cells (CAR-T cells) has significantly improved PFS and overall survival (OS) compared to the standard of care. However, outcomes remain poor in patients who fail or relapse after CAR-T cell therapy, and the optimal treatment strategy after CAR-T cell therapy failure has yet to be elucidated [4].

In this case report, we show the treatment sequencing of a patient relapsing after CAR-T cell therapy together with the kinetics of the CAR-T cells during relapse.

## Case presentation

**Figure 1:**
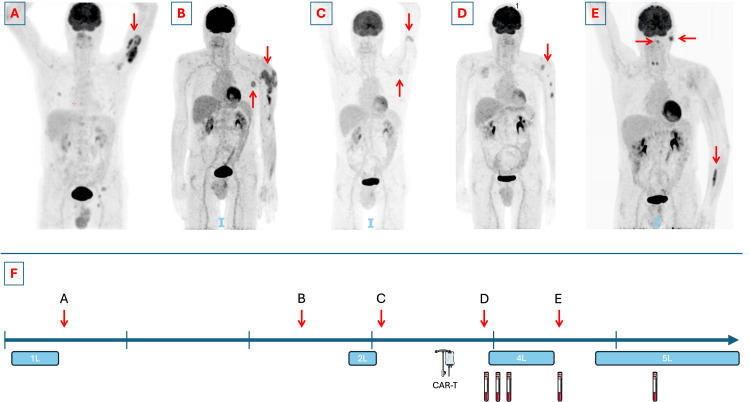
PET-CTs at diagnosis and relapses and treatment timeline Panel A: PET-CT at diagnosis (for clarity, only the most important lesions were marked). Panel B: PET-CT demonstrating first progression after first-line therapy. Panel C: PET-CT demonstrating progressive disease after second-line therapy. Panel D: PET-CT demonstrating progressive disease after CAR-T cell therapy. Panel E: PET-CT demonstrating progressive disease after fourth-line therapy. Panel F: Timeline demonstrating the disease course since diagnosis. Every vertical tick marks a year. Every red arrow is the time point of relapse, referring to the corresponding PET-CT with a letter. Treatment periods are marked with a blue box, labeled with the line (e.g., 1L is first-line treatment). Time points of CAR-T cell quantification are marked with a sample tube. PET-CT: positron emission tomography-computed tomography, CAR-T cell: chimeric antigen receptor T-cell This figure was drawn in part using images from Servier Medical Art. Servier Medical Art by Servier is licensed under a Creative Commons Attribution 4.0 Unported License (https://creativecommons.org/licenses/by/4.0/).

A 67-year-old male presented with persistent complaints of sinusitis for five months despite treatment with pseudoephedrine and a short course of systemic methylprednisolone. One month after the onset of symptoms, the patient developed an exophoria. A cerebral computed tomography (CT) scan was performed, demonstrating a soft tissue mass associated with osteolysis of the right maxillary sinus, with propagation to the lower nasal cavity and the hard palate. The lesion was biopsied at the maxillary sinus, and pathological examination of the biopsy showed a germinal center (GC) subtype DLBCL. An 18-fluoro-deoxy-glucose (FDG) positron emission tomography (PET)-CT scan confirmed a hypermetabolic lesion at the right maxillary sinus. Additionally, suspect soft tissue hypermetabolism of the left upper arm, without CT correlates, and suspect lesions at the interpolar region of the right kidney and additional multiple skeletal lesions were demonstrated (Figure 1A). Bone marrow aspirate and biopsy were suggestive of bone marrow invasion by the lymphoma. Cerebrospinal fluid analysis did not show central nervous system (CNS) invasion. The staging according to the Ann Arbor classification was stage IVA. The Revised International Prognostic Index (R-IPI) predicted a poor prognosis with a score of 3 points. Treatment was initiated with R-CHOP for a total of seven cycles along with two cycles of high-dose methotrexate (3,000 mg/m²), resulting in a complete response on PET-CT.

However, a PET-CT performed one year after treatment showed the appearance of multiple highly hypermetabolic subcutaneous soft tissue lesions and an intramuscular hypermetabolic focus in the distal left upper arm and a pathological lymph node in the left axilla (Figure 1B). A biopsy of one of the subcutaneous lesions demonstrated a relapse of the GC subtype DLBCL. The patient was subsequently treated with rituximab, gemcitabine, and oxaliplatin for four cycles, attaining a Deauville 2 complete response.

However, five months after completing second-line treatment, a new lymph node of 4 cm was palpable in the left axilla, and a clinically suspect cutaneous violaceous plaque with a diameter of 2 cm was observed on the left upper arm. The lymph node was biopsied, showing a second relapse of the DLBCL (Figure 1C). The patient was referred to a CAR-T cell therapy center to receive treatment with tisagenlecleucel (Kymriah®) (day 0) after lymphodepletion with fludarabine and cyclophosphamide (day -5 to -3). The treatment was complicated by a grade 1 cytokine release syndrome presenting with fever, treated with antipyretics. On day +30, a clinical regression of the cutaneous lesions was observed, and PET-CT showed a Deauville 2 response. Re-evaluation by PET-CT on day +90 showed evidence of a locoregional relapse with pronounced hypermetabolism of the cutaneous lesions (Figure 1D). An excision biopsy confirmed a cutaneous relapse of the DLBCL with about 30% positivity for CD19. Molecular quantification of the CAR-T cells was performed by an in-house optimized digital PCR (dPCR) for the detection and quantification of tisagenlecleucel CAR-T cells on day +136, showing detectable CAR-T cells, however at a very low level (less than 66.7 copies per microgram (µg) of DNA).

On day +89, the patient presented in the CAR-T cell therapy center with a left-sided peripheral facial paresis. Examination with CT and lumbar puncture (LP) did not show any evidence of invasion by the lymphoma. Magnetic resonance imaging (MRI) was not performed because of incompatibility with the pacemaker. Systemic treatment was started with corticosteroids, with a partial response in the paresis.

The patient was admitted to our hematology ward on day +130 because of a bilateral motoric neuropathy of the lower limbs. Repeat LP showed central nervous system invasion by the lymphoma, confirmed by cytomorphology and flow cytometric analysis. Treatment was started based on a phase 1b trial by Grommes et al. [5] with high-dose systemic methotrexate (3.5 g/m² every two weeks for a total of eight doses), corticosteroids, and rituximab, associating ibrutinib after methotrexate clearance. A new quantification of CAR-T cells was performed on day +150, but no CAR-T cells could be detected. The limit of detection (LOD) in this sample was 17.7 copies per µg of DNA. However, on day +184, quantification showed a moderate re-expansion of the CAR-T cell population with 125.8 copies per µg of DNA. Additionally, local treatment of the cutaneous lesions was performed with image-guided radiation therapy (IGRT) with a total of five fractions of 6 Gray. Eight cycles of rituximab and methotrexate were administered, along with continuous treatment with ibrutinib with a good clinical response.

**Figure 2:**
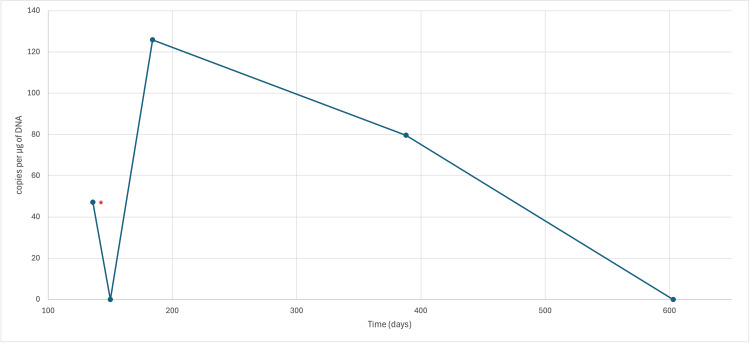
Evolution of CAR-T cell followed by dPCR The asterisk shows a value below the LOD. CAR-T cell: chimeric antigen receptor T-cell, dPCR: digital polymerase chain reaction, LOD: limit of detection, DNA: deoxyribonucleic acid

A post-treatment PET-CT was performed on day +388, showing heterogeneous FDG tracer uptake throughout the left arm, most pronounced in the muscles of the distal forearm, and the appearance of a new adenopathy of 2 cm in the left pre-auricular region and a hypermetabolic region in the left pharynx (Figure 1E). A muscle biopsy was planned but deferred due to familial circumstances. CAR-T cell quantification showed low-level persistence with 79.6 copies per µg of DNA. Excision biopsy of the lymph node confirmed a fourth relapse of the DLBCL, with examination of the cerebrospinal fluid also confirming a CNS relapse. Treatment with rituximab and lenalidomide according to the treatment schedule of the Rituximab Lenalidomide versus Any Chemotherapy (RELEVANCE) trial [6] was initiated. CAR-T cell analysis on day +603 failed to detect any CAR-T cells; the evolution of CAR-T cell quantification is visible in Figure 2. During the last follow-up on day +706, the patient was in good clinical condition with PET-CT confirming a complete response.

## Discussion

The treatment of R/R DLBCL has been revolutionized with the advent of CAR-T cell therapy, with an objective response rate of 53%, a median progression-free survival of 2.9 months, and a median OS of 11.1 months in third line [7]. However, real-world data, as published by Di Blasi et al. [4], showed that 43.3% of patients progressed or relapsed after CAR-T cell therapy after a median follow-up time of 7.9 months. The outcome of patients relapsing after CAR-T cell therapy is dismal, with a median OS of 5.2 months [4], and the optimal treatment strategy has yet to be elucidated. 

Multiple resistance mechanisms to CAR-T cell therapy have been identified. CD19-negative relapse has been described as the result of CD19 loss during CAR-T cell treatment due to selective pressure, disrupted CD19 cell membrane transport after the endoplasmic reticulum, CD19 mutations leading to a loss of the CAR-T cell binding epitope, or a preexisting CD19-negative population during treatment. Furthermore, CD19-positive relapses could be attributed to limited CAR-T cell expansion, CAR-T cell exhaustion, or T-cell senescence. Additionally, tumor cells could develop immune escape mechanisms including producing immunosuppressive cytokines (i.e., IL-10 or TGF-β) or presenting immunosuppressive receptors such as PD-L1 [8,9]. Interestingly, some retrospective studies showed that CD19 loss during treatment may be a less frequent resistance mechanism in DLBCL compared to B-cell acute lymphoblastic leukemia with only 6% of relapse samples showing a loss of CD19 [10].

Here, we describe a patient treated with tisagenlecleucel as third-line therapy presenting with an early failure after therapy with initially only extranodal disease in sanctuary sites (skin and central nervous system). He was treated successfully with an approach combining radiation, chemoimmunotherapy, and targeted treatment with the Bruton tyrosine kinase (BTK) inhibitor ibrutinib in the fourth line. BTK inhibitors have been attributed to activity in R/R DLBCL [11,12]. Additionally, BTK inhibitors have also been theorized to have a beneficial effect on CAR-T cell function by enhancing expansion, viability, and engraftment, and improving activation and effector function [13-15]. This could align with our findings of the moderate re-expansion of the CAR-T cell population during treatment with ibrutinib as shown above.

After his fourth relapse, the patient is still responding to fifth-line treatment with rituximab and lenalidomide, currently 12 months after starting treatment, despite experiencing a second CNS relapse. This treatment regimen has been approved as first-line therapy for advanced follicular lymphoma, showing comparable outcomes to rituximab and chemotherapy combination therapy [6]. Additionally, Gini et al. [16] investigated this combination in frontline settings for frail patients with newly diagnosed DLBCL not eligible for therapy with anthracyclines, showing moderate activity. This treatment was chosen in this particular case because it has been shown that lenalidomide passes the blood-brain barrier [17], and in the CAR-T cell setting, specifically, lenalidomide has been shown to mitigate T-cell exhaustion and stimulate CAR-T cell activation [10,18], leading to improved OS in monotherapy compared to chemotherapy regimens after CAR-T cell failure [4,10]. However, in our patient, we did not observe a new increase in CAR-T cells.

In our article, we presented a patient living with an R/R DLBCL with a complicated treatment course (Figure 1F), who relapsed after CAR-T cell therapy, along with the kinetics of the CAR-T cell population assessed by dPCR. Despite suffering two CNS relapses, after an early relapse after CAR-T cell therapy, he is currently still responding to his fifth-line therapy with a good quality of life. The limitations of this case report include that the time points of CAR-T cell quantification were not predefined, and no analysis was performed at the time of starting treatment with lenalidomide. However, it highlights the need for further research on the effect of immunomodulatory drugs on CAR-T cell expansion, survival, and efficacy.

## Conclusions

CAR-T cell therapy improves the outcomes in R/R DLBCL; however, a significant proportion of the treatment population may experience a relapse. In this population, outcomes are dismal, and there is an unmet medical need to improve survival. One of the approaches to improving outcomes after CAR-T cell therapy may be by harnessing the immunomodulatory effects of BTK inhibitors and lenalidomide. Further research is needed to identify the optimal treatment strategy after CAR-T cell therapy failure.
